# The role of lipids in genome integrity and pluripotency

**DOI:** 10.1042/BST20230479

**Published:** 2024-03-20

**Authors:** Qiyu Tian, Hoyoung Chung, Duancheng Wen

**Affiliations:** Ronald O. Perelman and Claudia Cohen Center for Reproductive Medicine, Weill Cornell Medicine, New York, NY 10065, U.S.A.

**Keywords:** culture medium, embryonic stem cells, genome integrity, lipids, pluripotency

## Abstract

Pluripotent stem cells (PSCs), comprising embryonic stem cells (ESCs) and induced pluripotent stem cells (iPSCs), offer immense potential for regenerative medicine due to their ability to differentiate into all cell types of the adult body. A critical aspect of harnessing this potential is understanding their metabolic requirements during derivation, maintenance, and differentiation *in vitro*. Traditional culture methods using fetal bovine serum often lead to issues such as heterogeneous cell populations and diminished pluripotency. Although the chemically-defined 2i/LIF medium has provided solutions to some of these challenges, prolonged culturing of these cells, especially female ESCs, raises concerns related to genome integrity. This review discusses the pivotal role of lipids in genome stability and pluripotency of stem cells. Notably, the introduction of lipid-rich albumin, AlbuMAX, into the 2i/LIF culture medium offers a promising avenue for enhancing the genomic stability and pluripotency of cultured ESCs. We further explore the unique characteristics of lipid-induced pluripotent stem cells (LIP-ESCs), emphasizing their potential in regenerative medicine and pluripotency research.

## Introduction

**Figure 1.**
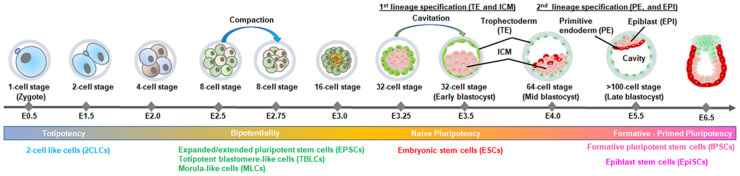
Preimplantation mouse embryonic development. The illustration depicts the sequence of events during preimplantation mouse embryo development, highlighting relevant embryonic stages and cell lineages that emerge following the first and second cell-fate decisions. Various types of stem cells have been derived from different embryonic stages. Stem cells originating from early embryonic stages (<E3.0) demonstrate bipotentiality.

**Figure 2.**
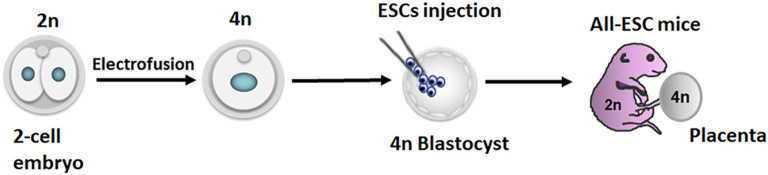
Tetraploid complementation scheme. The diagram illustrates the process of tetraploid complementation. Wild-type embryos at the two-cell stage are recovered and subjected to electrofusion, merging the two blastomeres into a single large blastomere, effectively doubling the DNA content from 2n to 4n. These 4n embryos are cultured *in vitro* to blastocysts. Subsequently, ESCs are injected into the 4n blastocysts, and the injected embryos are returned to the uterus for further development. In this process, host 4n cells predominantly contribute to the formation of the placenta but have limited involvement in the embryo proper. Conversely, ESCs play a significant role in the development of the embryo proper rather than the placenta. The resulting pups are derived entirely from ESCs.

Pluripotent stem cells (PSCs) not only provide a tool for studying early development but also hold tremendous therapeutic potential due to their ability to differentiate into all cell types of the body. Various types of PSCs have been successfully derived and maintained *in vitro*. These include embryonic stem cells (ESCs) typically derived from the inner cell mass (ICM) of blastocysts at embryonic day 3.5–4.5 (E3.5–E4.5) [1], formative pluripotent stem cells (fPSCs) from E5.5 to E6.5 embryos [2,3], and epiblast stem cells (EpiSCs) derived from post-implantation epiblasts between E5.5 and E8.25 [4,5] (Figure 1). In contrast, induced pluripotent stem cells (iPSCs) represent a groundbreaking achievement, where adult cells are reprogrammed to regain pluripotent capabilities, akin to ESCs, effectively circumventing ethical concerns associated with ESCs [6]. Recent studies have focused on isolating PSCs with bipotentiality from early preimplantation embryos, which can contribute to both the embryonic and extraembryonic lineages [7–12], aiming to recapitulate totipotency (Figure 1). In this review, we consider all these PSCs that are derived from preimplantation embryos as ESCs. In mice, the pluripotency of ESCs is demonstrated by the ability to produce entirely ESC-derived mice (termed all-ESC mice) with the aid of a tetraploid (4n) embryo (Figure 2), a capability known as 4n-competency [13,14]. However, despite significant advancements in the isolation and expansion of ESCs *in vitro*, the lack of robust and reliable culture systems poses challenges for both the derivation and long-term maintenance of ESCs, as well as their application in regenerative medicine [15–19].

Pluripotent mouse ESCs (mESCs) were initially established and cultured in medium supplemented with fetal bovine serum on feeder layers of mouse embryonic fibroblasts. However, undefined serum components lead to heterogeneous cultures and a gradual loss of pluripotency [20]. The breakthrough discovery that inhibition of Mek1/2 and Gsk3β (2i) maintains ESCs in a more homogeneous state of naive pluripotency [21] allowed stabilization and expansion of ground-state ESCs *in vitro* in medium supplemented with LIF*.* This defined serum-free 2i/LIF medium is now widely used in stem cell research. It enables the derivation of ESC lines from non-permissive mouse strains and other mammalian species that previously failed in ESC derivation using serum-containing media. However, prolonged culture of mESCs in 2i/LIF [15,16] or human ESCs in 5i/LIF + Activin medium [22], unfortunately, causes aneuploidy, DNA hypomethylation, and loss of imprinting, impairing developmental potential. Such detrimental effects are even more severe with female ESCs, and the 2i/LIF system does not support the derivation of fully potent female ESC lines [15,16].

We recently discovered that adding lipid-rich albumin, specifically AlbuMAX (AX), to the standard 2i/LIF medium can significantly improve the genome stability and developmental potency. This addition also induces a pluripotency transition from naive to a formative-like state and enhances responsiveness to differentiation clues in mESCs [23,24]. We further demonstrated that it is the lipids, rather than the albumin in AX, that are responsible for these effects [23,24]. Similar to naive 2i/LIF-ESCs, AX-ESCs (ESCs cultured in AX-containing 2i/LIF medium) can generate all-ESC mice but possess distinct properties compared with both naive 2iL-ESCs and reported formative PSCs (fPSCs) [2,3]. These properties include full developmental potency, long-term genomic stability, heightened responsiveness, and bipotentiality. Thus, AX-ESCs represent a new type of ESCs, termed *lipid-induced pluripotent* ESCs (LIP-ESCs).

This review aims to provide an overview of latest advancements in understanding the roles of lipids in genome stability and pluripotency in ESCs. Lipids constitute a diverse group of molecules, encompassing fatty acids, phospholipids, sphingolipids, sterols, and lipid-derived signaling molecules [25]. Traditionally recognized for their role in maintaining cellular homeostasis, serving as an energy source through mitochondrial fatty acid oxidation, facilitating intracellular signal transduction, and contributing to macromolecules essential for membrane biosynthesis during cellular growth and proliferation [25,26]. Beyond their traditional functions as structural components of cellular membranes and energy storage molecules, recent studies suggest that lipids also play vital roles in regulating genome stability and pluripotency in ESCs [24,27,28].

## Lipids play a vital role in maintaining the genome stability of ESCs

### Lipids maintain nuclear homeostasis

Lipids play a pivotal and apparent role in nuclear homeostasis, primarily serving as structural components of the nuclear membrane. Each cellular membrane establishes its unique identity through a distinct composition of proteins and lipid species, influencing various membrane attributes, including curvature and electrostatic characteristics. Lipids that introduce significant curvature to the membrane create an environment conducive to attracting specific proteins [25,29]. The lipid composition of the nuclear membrane significantly shapes the assembly and configuration of nuclear pore complexes [30]. A significant contribution is attributed to the sphingolipid hydrolase SMPD4 [31], which, by releasing ceramides specifically in proximity to nuclear pores, may either promote the local concave membrane curvature required for the insertion of nuclear pore complexes [32] or acting as enzymatic cofactors. Additionally, the production of very-long-chain fatty acids (FAs) has been found to prevent ruptures in the fission yeast nuclear membrane [33], essential for sustaining the extreme curvature of the membrane at nuclear pore complex insertion sites [34]. Lipids also help alleviate stress resulting from defects during nuclear pore complex assembly [35], and the overexpression of *Ole1*, which increases the presence of unsaturated acyl chains, resolves defects in nuclear envelope sealing [36]. The nuclear membrane lipid profile also supports its role in genome shielding. For example, the accumulation of long-chain sphingoid bases suppresses the aberrant nuclear membrane defects induced by aneuploidy both in budding yeast and human cells [37]. This alleviation occurs through membrane deformation or the emission of membrane-derived structures into the nucleoplasm, processes that can impact ploidy control or DNA repair, respectively [38,39].

### Lipids influence the epigenome of ESCs

Epigenetics, representing heritable changes in gene expression without alterations to the underlying DNA sequence, introduces another dimension to genome stability. Some lipids serve as precursors (PEs) or modulators for molecules that are critical for epigenetic modifications. For instance, acetyl-CoA forms a well-established bridge between energy metabolism and chromatin regulation [40]. *De novo* FA synthesis utilizes acetyl-CoA as its substrate. This directly competes with the requirement of acetyl-CoA for histone acetylation. Consequently, decreasing the rate of FA synthesis by lowering acetyl-CoA-carboxylase (ACC1) expression enhances global histone acetylation and gene expression [41]. Conversely, stimulating FA oxidation, which releases acetyl-CoA, produces a similar effect [42]. Hence, both physiological and pathological changes in mitochondrial activity swiftly impact the epigenome [43]. This extends to tricarboxylic acid (TCA) intermediates like acetyl-CoA and α-ketoglutarate, as they serve as substrates for histone acetyltransferases and demethylases, respectively [27,44].

DNA hypomethylation is widely recognized to induce genomic instability by facilitating chromosomal rearrangements [45,46]. The 2i/LIF culture system, which involves the inhibition of Mek1/2 and Gsk3β (2i), is used to culture and maintain ESCs in a more homogenous naive ground state [21]. However, extended Mek1/2 suppression of ESCs in this medium leads to aneuploidy, global DNA hypomethylation and loss of imprinting. This is typically more prominent for female ESCs, leading to rapid loss of pluripotency in early passages [15,16,23,24]. Our recent study has shown that supplementing the 2i/LIF medium with AX, can mitigate DNA hypomethylation and prevent loss of imprinting, thereby supporting the long-term culture of mESCs while preserving pluripotency [23,24]. In 2i/LIF cultures, the DNA methyltransferase 3 enzymes (DNMT3A, DNMT3B, DNMT3L), which add a methyl group to the carbon 5 position of cytosine residues in DNA — a process known as DNA methylation — are suppressed in ESCs. However, we found that the expression levels of these genes significantly increase when lipids are introduced to the 2i/LIF medium [23,24]. This observation is consistent with findings in human PSCs cultured in an AX-supplemented medium [27]. Lipids modulate the expression of DNMT3s, affecting DNA methylation and consequently reshaping the global epigenome of ESCs, which influences genome stability. However, the mechanism by which specific lipid species regulate DNMT3s’ expression in ESCs remains elusive.

### The role of lipids in regulating nucleotide pools in ESCs

Endogenous nucleotide pool imbalances can adversely affect DNA replication and repair processes, potentially leading to genome instability [47–49]. In our studies, we found a pronounced reduction in the steady-state endogenous nucleotide pools of ESCs when cultured in 2i/LIF medium. There was a noticeable decline in levels of nucleotides such as thymidine, 2′-deoxyguanosine, and 2′-deoxyinosine, as well as their precursor, aspartate [23,24]. Meanwhile, the nucleotide biosynthesis pathways are impacted in 2i/LIF medium, as the expression of Prps2, an ATP-dependent enzyme in the syntheses of purines and pyrimidines from ribose 5-phosphate [50], is significantly down-regulated in ESCs cultured in 2i/LIF medium [23,24]. Interestingly, when lipids were introduced to the 2i/LIF medium, there was a marked increase in Prps2 expression, leading to an increase of the endogenous nucleotide pools [23,24].

Nucleotide pool depletion is widely recognized as a factor that contributes to genome instability [46–49]. The effects of such depletion are multifaceted and cumulative, impacting DNA replication, repair processes, and telomere shortening [47,48,50,51], collectively contribute to genome instability. Supplementing the 2i/LIF medium with lipids mitigates the suppression of nucleotide biosynthesis pathways, subsequently promoting genome stability in ESCs. Further investigation is warranted to explore the link between lipids and nucleotide pools.

### Lipids and their role in mitigating aneuploidy and X chromosome loss in ESCs

ESCs cultured in 2i/LIF medium exhibit high levels of aneuploidy, notably trisomy and X chromosome loss, especially in female ESCs [15,16,24]. In our study, ∼95% of the cells in 2i/LIF medium display aneuploidy by passage 15, with trisomy 8 being the most prevalent. By passage 43, nearly all cells show aneuploidy, with a significant proportion exhibiting autosomal chromosome loss [23,24]. In contrast, ESCs cultured in 2i/LIF medium supplemented with lipids are more likely to maintain a normal karyotype [23,24].

Female ESCs cultured in 2i/LIF medium tend to lose one of their two X chromosomes [15,16]. Our study using a dual reporter female ESC line (X^GFP^X^Tomato^) [15] and flow cytometry analysis revealed that ∼40% of cells at passage 10, and over 90% at passage 15, lost one X chromosome in 2i/LIF medium. In contrast, ESCs in lipid-supplemented 2i/LIF medium maintained two X chromosomes in over 95% of the cells with minimal fluctuation between passage 3 and passage 15 [23,24]. This suggests that lipids can efficiently prevent X chromosome loss in female ESCs and promote karyotypic stability in ESC cultures. However, the specific mechanisms by which lipids mitigate X chromosome loss remain unknown and require further elucidation through comprehensive research and experimentation.

## Lipids regulate the self-renewal and pluripotency of ESCs

### Lipid metabolism and ESCs

Lipids and FAs play a crucial role in energy storage and metabolism, influencing ESC proliferation and differentiation [51]. They can function as an energy reserve or a source of energy through β-oxidation, impacting the pluripotent state and maintaining the balance between glycolysis and oxidative phosphorylation. Additionally, diapause, a reversible state of ESC proliferation and developmental progression, may be linked to metabolism. The Tfcp2l1-Cpt1a-FAO (FA oxidation) axis has been identified as promoting the survival of quiescent ESCs and diapause-like blastocysts under metabolic stress [52]. Moreover, intracellular acetyl-CoA accumulation, resulting from reduced *de novo* FA synthesis and increased FA β-oxidation, enhances endoderm differentiation in hESCs through SMAD3 acetylation [53]. Conversely, FA synthesis may regulate ESC pluripotency. Mitochondrial dynamics and mitochondrial DNA are emerging as crucial regulators in maintaining pluripotency and cell reprogramming [54,55]. ACC1, a lipogenic enzyme, has been reported to regulate *de novo* FA synthesis and promote mitochondrial fission in both ESCs and iPSCs, contributing to the maintenance of the pluripotent state of stem cells [56].

### Lipids regulate the self-renewal of ESCs

One of the fundamental properties of ESCs is their capacity for self-renewal. Although significant advances have been made in designing chemically defined media that support the self-renewal and proliferation of ESCs *in vitro*, the media available for ESC culture remain unsatisfactory. In their pursuit to develop chemically defined media that robustly sustain ESC self-renewal, Garcia-Gonzalo and Belmonte [57] discovered that a commercially available serum replacement product enhanced the growth of undifferentiated hESCs when added to an established chemically defined medium. Subsequent experiments indicated that this beneficial effect stemmed from the lipid-rich albumin component, AX, within the serum replacement. Intriguingly, this activity was trypsin-resistant, suggesting that the lipids, not the albumin itself, were driving the effect. This hypothesis was further supported when lipid-poor albumin exhibited no discernible activity. While Garcia-Gonzalo and Belmonte's study identified the primary lipids within this lipid-rich albumin, pinpointing the exact lipid or combination of lipids in AX that influence ESC self-renewal remains a challenge. It is plausible that a synergistic effect from multiple lipids in AX plays a role, rather than a single lipid or a few of them. Corroborating this observation, our recent study demonstrated that AX can significantly enhance the proliferation of mESCs when cultured in a 2i/LIF medium. The doubling time of ESCs cultured in AX is just 6.7 h, compared with 10.2 h in 2i/LIF medium [23,24].

### Lipids induce pluripotency transition

Using the well-established, chemically defined 2i/LIF mESC culture system, we explored the impact of lipids on ESC pluripotency. We observed that when AX was introduced into the 2i/LIF medium (referred to as AX-ESCs), mESC colonies no longer displayed the typical uniform and rounded naive colony morphology. Analyses of the core pluripotency genes, Pou5f1 (Oct3/4) and Sox2, via immunostaining, qPCR, and western blotting, revealed that these genes are expressed at similar levels regardless of the culture medium. However, genes identified as naive markers, such as Nanog, Rex1 (Zfp42), Klf2, Klf4, Prdm14, Stat3, Essrb, and Nr0b1, showed reduced expression in AX-ESCs. In contrast, many formative pluripotency markers [2,3], including Wnt8a, Pou3f1, Pim2, Sall2, Sox3, c-Myc, ERas, and Lef1, were significantly increased in AX-ESCs. Genes exclusively associated with primed stem cells remained unexpressed in both 2i-ESCs and AX-ESCs [23,24]. As AX-ESCs can contribute to the germline and retain the potential to generate all-ESC mice through tetraploid complementation, they are not yet transformed to a primed state, but are likely maintained in a distinct naive-to-primed intermediate state of pluripotency. Our findings indicate that AX is able to facilitate pluripotency transition in mESCs.

To determine whether lipids are the active components of AX responsible for pluripotency transition, we removed proteins, including albumin, to create deproteinized AX (dep-AX). ESCs in 2i/LIF medium with dep-AX exhibited formative colony morphology, while those with FA-free BSA maintained naive colony morphology. Introducing chemically defined lipid components (CDLCs) into 2i/LIF resulted in changes to colony shape, albeit less pronounced than with AX. This suggests that lipids, not albumin, influence the colony morphology of AX-ESCs. Our study also revealed that lipids increase the expression of formative genes and decrease that of naive genes. Specifically, in media with AX or certain FAs, expression levels of formative genes increased, whereas naive genes decreased. Our results further confirmed the role of lipids in promoting pluripotency transition to a formative-like state, independent of BSA [23,24].

To explore whether lipids or lipid metabolism drive pluripotency transition, we inhibited carnitine palmitoyltransferase (CPT1) using Etomoxir (ETO), an irreversible inhibitor of CPT1 located on the inner face of the outer mitochondrial membrane. When we supplemented 2i/LIF medium with AX and ETO, a pluripotency transition from the naive to the formative-like state was still observed. ACAA2 is responsible for the final step in the mitochondrial FA β oxidation spiral. Notably, inhibiting ACCA2 with Trimetazidine (TMZ) also failed to block this pluripotency transition. In line with the inhibitor, the Cpt1a null ESCs exhibited a formative-like colony morphology in the 2i/LIF medium supplemented with AX and showed increased expression of many formative genes [23,24]. These findings suggest that FA oxidation is dispensable for lipid-induced pluripotency transition, indicating that lipids themselves, not their metabolism, drive the transition.

FAs have been recognized as substrates for the acylation of proteins for differentiation and pluripotency [58]. An inhibitor targeting the enzyme SCD1 (Stearoyl-coenzyme A desaturase 1) — responsible for converting saturated FAs into monounsaturated FAs (MUFAs) — aids in preserving the primed pluripotent state of hPSCs by counteracting the onset of endodermal differentiation. The combination of oleate and the SCD1 inhibitor effectively restores endodermal markers. This is supported by data showing that hPSCs treated with the inhibitor undergo reduced protein acylation during differentiation and display changes in the lipid-regulated Wnt/β-catenin pathway [59].

Cornacchia et al. [27] associated lipid supplementation with hPSC differentiation. Under lipid-free conditions (E8), hPSCs experienced significant epigenetic changes, including DNA hypomethylation and the up-regulation of pluripotency markers that correlated with an intermediate naive-to-primed pluripotent state. However, these characteristics were lost upon the addition of lipids. The authors suggested that these changes were triggered by a high requirement for *de novo* lipogenesis in the lipid-free E8 medium.

A recent study [28] explored the components of AX, revealing that free FAs or cholesterol present in the medium relieve the requirement for *de novo* lipogenesis but do not induce any additional metabolic shift. Rather, the metabolic and transcriptional landscape of conventional hPSCs cultured in the presence of AX is modulated by lysophosphatidic acid (LPA), a hydrophilic lipid with various functions for signaling pathways. LPA is known to promote the differentiation of conventional hPSCs into neuroepithelial rosettes via SRF (serum response factor) signaling [59,60]. LPA treatment induces an accumulation of metabolites of glycolysis, the pentose phosphate pathway, and the TCA cycle, accompanied by altered mitochondrial morphology and functionality. LPA causes cell morphology changes, with bigger and flatter colonies and reduced proliferation. Notably, such changes are similar to those induced by AX and are all reversible. In contrast with previous findings by Cornacchia et al. [27], the study by Xu et al. [28] excludes the involvement of excessive *de novo* lipogenesis in the cellular state observed in E8, as the provision of exogenous FAs and cholesterol fails to produce any significant change. Moreover, the removal of LPA does not recapitulate the naive-to-primed state described by Cornacchia et al. [27]. These studies, along with our findings, suggest a role for lipids in inducing the pluripotency transition of ESCs.

### Lipids sustain pluripotency states by interacting with signaling pathways

**Figure 3.**
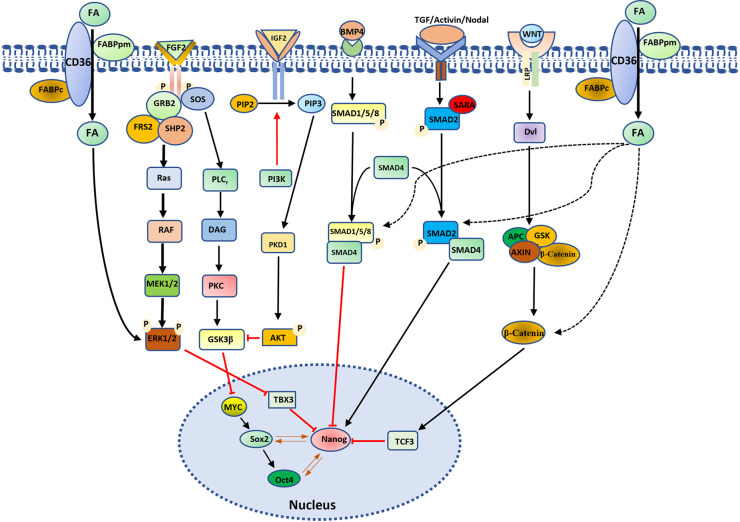
Schematic illustration of the interaction of the lipid pathway with other signaling pathways for the regulation of pluripotency. Lipids (FA, fatty acid) enhance ERK activity by increasing ERK phosphorylation (p-ERK elevation). The ERK signaling pathway, in turn, modulates TBX3 expression, where TBX3 acts as a transcriptional regulator for Nanog expression. Lipids can also engage with additional signaling pathways that impact Nanog expression, thereby influencing the pluripotency state of ESCs.

Lipid signaling pathways are pivotal in maintaining the delicate balance between pluripotency maintenance and stem cell differentiation. Lipid-derived signaling molecules, including prostaglandins, sphingolipids, and phosphoinositides, serve as potent regulators of pluripotency-related transcriptional networks by modulating key signaling pathways such as PI3K-Akt, Mapk-Erk, and Wnt [61–63]. These pathways exert influence over the expression and activity of pluripotency-associated transcription factors, such as Oct4, Sox2, and Nanog, as well as lineage-specific transcription factors, orchestrating the transition between pluripotent and differentiated states [24,56,64,65]. In a study involving hPSCs, Cornacchia et al. [27] discovered that the addition of lipids to the E8 medium induced a transition of hPSCs from an intermediate formative pluripotent state to a primed state. Our study in mESCs demonstrated that lipids induce mESCs to exit from the naive state and maintain a formative-like state, rather than a primed state [23,24]. This suggests that lipids play a crucial role in ESC differentiation, and the pluripotent state is determined by the interaction and balance between lipids and other signaling pathways that promote the naive pluripotent state, such as the inhibition of the Mapk-Erk signaling pathway by small molecules [21]. Our findings revealed a rapid response of Erk1/2 to the addition of AX, leading to elevated p-Erk1/2 levels [23,24]. This observation suggests a potential influence of lipids on Erk2 phosphorylation, contributing to the modulation of pluripotency transition. Nanog, a critical factor for maintaining stem cell pluripotency, serves as a regulatory target for multiple signaling pathways (Figure 3) [61–63]. Knockdown of Nanog has been demonstrated to induce ESC differentiation into primed cells in serum/LIF medium [66]. In our study, both transcript and protein levels of Nanog were down-regulated in AX-ESCs [23,24]. Our results propose a model in which lipids promote Erk signaling to down-regulate Nanog, while Fgf/Erk inhibitors suppress Erk activity, leading to the up-regulation of Nanog. Consequently, the pluripotency state in ESCs is intricately linked to the Nanog level (Figure 3).

### Lipids induce bipotentiality of mESCs

Following fertilization, the development of a totipotent zygote goes through several intermediary stages that involve asynchronous cell divisions without altering cytoplasmic volume. This results in smaller cells called blastomeres, which form a blastocyst capable of implantation into the uterus [67]. The blastocyst features an outer epithelial layer of trophoblasts (TE) that serves as a precursor to the embryonic component of the placenta, and a fluid-filled cavity with an ICM that results from the first lineage specification. The ICM undergoes a second lineage specification, giving rise to differentiating epithelial cells that serve as yolk sac PEs and the pluripotent epiblast (EPI) cells that constitute the entire embryo (Figure 1). At the two-cell (2C) stage, mouse blastomeres are totipotent, such that separated 2C stage blastomeres can produce normal twin pups [68,69]. Totipotency decreases at the 4C stage and is completely lost at the 8C stage [67–70]. Liveborn offspring cannot be generated from a single 8C stage blastomere without accompanying carrier blastomeres. However, 4C and 8C blastomeres are bipotential, as an individual blastomere can contribute to both embryonic and extraembryonic lineages. ESCs were initially derived from ICM cells in blastocyst stage, which can differentiate into all cell types in an adult body. While it was believed that they lost bipotentiality, recent reports have shown that ICM cells retain their bipotential nature until the second lineage specification [71,72].

ESCs cultured in serum/LIF medium contain a subset of 2-cell-like cells (2-CLCs) capable of contributing to both embryonic and extraembryonic lineages within a chimeric embryo [73]. These 2-CLCs exhibit bipotentiality, characterized by transient expression of both Zscan4 and MERVL [73]. In our studies, we observed a significant up-regulation of Zscan4 in AX-ESCs compared with 2i-ESCs [23,24]. Using *Zscan4*-GFP and *MERVL*-tdTomato double reporter ESCs designed to detect transiently expressed early embryonic transcripts Zscan4c [74] and *MERVL* endogenous retrovirus [73], we found that AX-ESCs present a higher percentage of double-positive cells than 2i-ESCs [23]. Therefore, AX-ESCs exhibit an enhanced capacity for bipotentiality.

While the expanded 2-CLCs population in AX-ESCs might account for their bipotentiality, their heightened responsiveness to inductive cues for lineage specification offers another explanation for AX-ESCs’ bipotentiality. Using a directed differentiation protocol, we found that AX-ESCs can rapidly respond to Activin A induction to differentiate into mesoderm. In comparison, naive 2i-ESCs necessitate a 2-day preparatory period, during which they form embryoid bodies, before responding to Activin A induction [23,24].

Injecting ESCs into 8C stage embryos (E2.5) is a widely accepted standard assay for testing bipotentiality of PSCs [71–73]. Notably, the initial lineage specification into TE and ICM in embryos takes place at the 32C stage, occurring at E3.5 (Figure 1). This progression from the 8C stage to the 32C stage spans just 1 day (Figure 1). When introduced into an 8C stage embryo, AX-ESCs have the capability to respond to differentiation cues and transform into TE. However, naive 2i-ESCs are non-responsive during this window. As a result, in the chimeric assay, AX-ESCs can contribute to the TE lineage, while naive 2i-ESCs cannot.

## Conclusions

**Figure 4.**
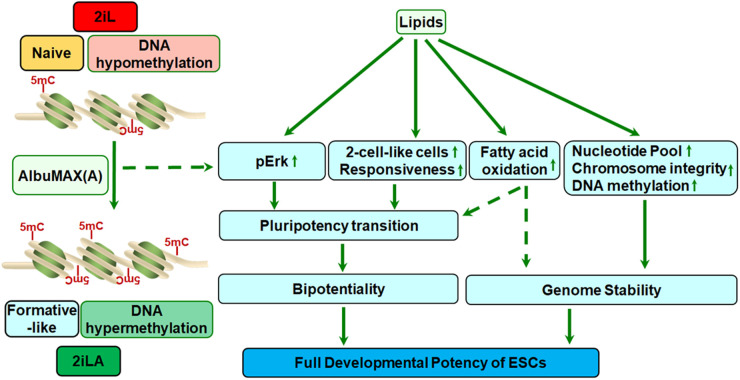
Schematic illustrating the dual role of lipids in genome stability and pluripotency of embryonic stem cells (ESCs). ESCs cultured in 2i/LIF (2iL) medium exhibit naive pluripotency and undergo DNA hypomethylation. The addition of AlbuMAX, a lipid-rich albumin, to the 2iL medium (2iLA) leads to an increase in DNA methylation and triggers a pluripotency transition by stimulating Erk phosphorylation. Lipid metabolism reduces lipogenesis, increases amino acid biosynthesis and non-canonical TCA metabolites, all of which drive the transition from naive pluripotent state to a formative-like state. Lipids enhance the 2-CLC population and promote ESC responsiveness to specification cues, inducing ESC bipotentiality. Lipids also up-regulate the expression of ZSCAN4, DNMT3s, and Prps2, essential genes for maintaining telomere length, DNA methylation, and nucleotide pools, which together enhance genome stability. Furthermore, lipids alleviate X chromosome loss and ESC aneuploidy, helping to preserve a normal karyotype. In essence, the dual role of lipids in both genome stability and pluripotency plays a pivotal role in maintaining the full developmental capacity of ESCs.

Stabilizing pluripotency in both male and female ESCs during long-term culture benefits research into the mechanisms of cell fate determination, epigenetic reprogramming, and modeling of early development. We have summarized the recent progress on the role of lipids in genome stability and pluripotency of ESCs. Lipids play a vital role in genome stability by providing nuclear structure, influencing the epigenome, mitigating metabolism, and maintaining a normal karyotype. Lipids also regulate the pluripotency of ESCs by directly stimulating Erk2 phosphorylation, driving the pluripotency transition. Lipid metabolism reduces lipogenesis and amino acid biosynthesis while promoting non-canonical TCA metabolites, which have recently been reported to be involved in pluripotency transition [75]. Furthermore, lipid metabolism promotes nucleotide and acyl-CoA biosynthesis, enhances the expression of ZSCAN4 and DNMT3s, and thereby improves genome stability during extended culture periods (Figure 4).

Lipids exert profound impacts on ESCs both genetically and epigenetically. These impacts are characterized by long-term genome stability, robust developmental potency, bipotentiality, and a pluripotent state distinct from ESCs cultured in lipid-free media. We propose to term these ESCs as lipid-induced pluripotent ESCs (LIP-ESCs). Due to their enhanced developmental potency and long-term genome stability, LIP-ESCs could offer a broad array of applications that can significantly benefit research in cell fate determination, epigenetic reprogramming, and early developmental modeling. The enhanced genome stability and potency of LIP-ESCs could lead to more efficient gene editing and genome engineering. This has considerable implications for personalized medicine, regenerative therapies, and the generation of genetically modified mouse models.

In summary, these findings underscore the importance of lipids in cell culture media for maintaining genomic, epigenomic, and phenotypic integrity and provide novel insights into developing optimal culture systems for ESCs.

## Perspectives

- Lipids play vital roles in the maintenance of cellular homeostasis by serving as an energy source through mitochondrial fatty acid oxidation, enhancing intracellular signal transduction, and providing macromolecules for membrane biosynthesis during growth and proliferation.
- Recent research underscores the significance of lipids in the genome stability and pluripotency of ESCs. These studies have shown that lipids can trigger a pluripotency transition, promote genome stability, and enhance the developmental potency of ESCs.
- The mechanisms by which specific lipids regulate genome stability and pluripotency in ESCs, as well as their interactions with other signaling pathways, remain unclear. Gaining insight into these questions will aid in designing optimal culture systems for ESC isolation.

## Abbreviations

2i-ESCs: ESCs cultured in 2iL medium
2iL: 2i + LIF
2iLA: 2i + LIF + AlbuMAX
A: AlbuMAX
AX-ESCs: ESCs cultured in 2iLA medium
CDLCs: chemically defined lipid components
EpiSCs: epiblast stem cells
ESCs: embryonic stem cells
fPSCs: formative pluripotent stem cells
hESCs: human embryonic stem cells
LIP-ESCs: lipid-induce pluripotent ESCs
PSCs: pluripotent stem cells
